# The Properties, Modification, and Application of Banana Starch

**DOI:** 10.3390/polym14153092

**Published:** 2022-07-29

**Authors:** Herlina Marta, Yana Cahyana, Mohamad Djali, Giffary Pramafisi

**Affiliations:** 1 Department of Food Technology, Faculty of Agro-Industrial Technology, Universitas Padjadjaran, Bandung 45363, Indonesia; y.cahyana@unpad.ac.id (Y.C.); djali@unpad.ac.id (M.D.); 2 Research Collaboration Center for Biomass and Biorefinery between BRIN and Universitas Padjadjaran, Bandung 45363, Indonesia; 3 Department of Agroindustry Technology, Lampung State Polytechnic, Bandar Lampung 35141, Indonesia; giffarypramafisi@polinela.ac.id

**Keywords:** banana starch, properties, modification, application

## Abstract

Banana is a tropical fruit crop that is consumed at large, not only because of the quantity produced but also because it serves the calorific needs of millions of people. Banana is a potential source of high starch content (more than 60%). The application of starch for various purposes is dependent upon its structural, physicochemical, and functional properties. A native starch does not possess all required properties for specific use in the food product. To improve its application, starch can be modified physically, chemically, and enzymatically. Each of these modification methods provides different characteristics to the modified starch. This review aims to examine the chemical composition, granule morphology, crystallinity, pasting, thermal properties, and digestibility of banana starch, and discusses the various modifications and potential applications of banana starch in the food industry.

## 1. Introduction

Banana (*Musa* sp.) is a widespread tropical fruit crop, largely consumed in the world; it consists of two major subgroups: banana and plantain. Both have high production and sources of calories that are required for millions of individuals, particularly across Asia and Africa. The term “banana” is used to extensively mean the dessert form and certain cooking types, while “plantain” is used for starchy bananas that are regularly cooked or processed [1,2].

Based on their genomic synthesis, palatable bananas are classified into 3 types: diploid (AA and AB), triploid (AAA, AAB, and ABB), or tetraploid (AAAA, AAAB, and AABB). Nonetheless, most pertinent bananas are triploid and are obtained from the wild species *Musa acuminata* (A) and *Musa balbisiana* [3,4]. Generally, dessert banana cultivars are AA or AAA and cooking bananas (plantains) are prevalently AAB, ABB, or BBB. The incredible biodiversity of banana plants provides potential for different utilizations and applications [5].

The chemical composition of bananas depends on the variety and ripening state; however, climatic conditions, agronomic traits, and type of soil also alter the major and minor components of banana. A previous study reported that unripe banana pulp has a lower moisture content than ripe banana pulp, and the carbohydrate content was higher in the unripe banana sample than in a ripe banana, but the opposite was true for fiber content [5]. Furthermore, Lii et al. [6] discovered that the starch encounters important changes during ripening. The chemical composition of bananas changes during the different stages of maturation: the starch degrades and the sucrose content increases.

Native banana starch has higher resistant starch content (65–98%) [7,8,9] than other native starches such as aracca starch (17.5%), cassava starch (1.8%), cush-cush yam starch (55.8%), potato starch (48.5%), and taro starch (13.8%) [10]. The high resistance of native banana starch to hydrolyze by α-amylase causes it to have a low glycemic index and makes it good for diabetic patients.

Generally, the application of bananas is still limited as a raw material for traditional foods. Banana has the potential to be processed into semifinished products such as starch or flour, which has several advantages: it is easier to apply and has a longer shelf life than the fresh form. Banana flour has the potential to replace wheat flour as a raw material in the manufacture of various bakery products such as bread, cookies, cakes, pasta, and others.

Native starch has several poor characteristics such as low thermal stability, creation of hard-formed gel, and creation of too-sticky pasta [11], making its applications limited in the food industry. To improve its characteristics, native starch must be modified so that it is more widely used as an ingredient in food products. The modifications alter the viscosity, association behavior, gel stability in final products [12], and the structure and the hydrogen bonding of native starch [13]. The change of starch properties occurs at the molecular level, with little or none later taking place in the superficial appearance of the starch granule [13]. One of the purposes of starch modification is to increase the thermal stability of starch pasta during heating and stirring, and to extend their application for many food industrial applications [12].

With the increasing demand for starch by the food industry, banana starch may be an alternative conventional starch source, which is used for various food industry applications. To support the current information on banana starch, a review is required to summarize various aspects of banana starch, i.e., extraction, chemical composition, structure, physicochemical properties, digestibility, modification, and potential applications in food products.

## 2. Banana Starch Extraction and Chemical Composition

Banana in a mature green stage is a great source of starch. The green banana pulp contains up to 70–80% starch on a dry weight basis [4]. A wet milling process is suitable for banana starch isolation due to low-level impurities [14]. Several techniques for starch isolation have been reported, such as alkaline [15,16] and non-alkaline extraction [17,18].

Zhang et al. [4] discovered that ripening stages and banana cultivars are the primary factors that affect the yield. Chávez-Salazar et al. [18] reported the extraction yield of banana starch of 5.78–12.73% and a total starch content of 74.9–84.0%. The starch consists substantially of carbohydrates; however, non-starch components such as lipids, proteins, and ash are present in the composition [19,20]. High purity of starch or low content of non-starch components indicates the best commercial quality of starch.

The chemical composition of starch extracted from various types of bananas and plantains is presented in Table 1. Starch extracted from banana has 6.83–14.00% moisture content and 0.03–2.08% ash. It also contains 0.01–2.46% and 0.17–2.16% lipid and protein, respectively. The difference in lipid and protein content of the starch might be caused by the different variety of banana and the different extraction technique that was used to extract the starch. The total starch ranged from 69.39% to 98.10%. The crude fiber and amylose contents of banana starch are 0.18–0.0.47% and 13.36–42.07%, respectively. As for the plantain, the amylose content of the starch is 22.76–38.79%.

## 3. Starch Properties

### 3.1. Granule Morphology

Generally, banana starch granules from various banana varieties are irregular, elongated, and round/spheroidal in shape [16,19,20,24,29]. Variety affects the granule shape of banana starch [19,20,22]. Furthermore, Marta et al. [20] reported that the elongated shape is mainly found in the granules of Kapas cultivars, while the round shape is mostly found in the granules of Kepok, Ambon, and Nangka cultivars.

**Figure 1:**
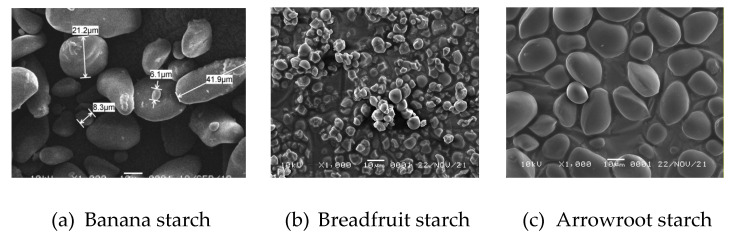
SEM images of native banana, breadfruit and arrowroot starches (1000× magnification).

The granule size of banana starch depends on the cultivar and the ripening state [18]. Based on the results of analysis using SEM, the size of starch granules from four banana cultivars varied (5.5–59 μm) [20], 3.33–56.66 μm [30], 18–50 μm [22], 6–80 μm and mostly between 20–60 μm [4], 10–80 μm and mostly between 30–50 μm [24]. The granule size of banana starch is larger than other starch, such as breadfruit starch (<10 μm) [31], wheat starch (2–20 μm) [32] and sorghum starch (4–35 μm) [33] (Figure 1).

### 3.2. Crystallinity

Starch granules are semicrystalline crystals that can be determined by an X-ray diffractometer (XRD). Several previous studies have reported that native banana starch exhibits various types of crystal structures, such as type-B [9,20,22,24,34], type C [16,35], and type A [36]. The crystallinity of banana starch is not clear. The type of crystal structure depends on the banana cultivar, plant growth conditions, isolation techniques and other factors [7,19,20,24]. Marta et al. [20] have discovered that three cultivars of cooking banana type demonstrated type B crystalline structure, i.e., Kapas, Kepok, and Nangka, while dessert banana has a type C crystalline structure, i.e., Ambon.

The crystalline type and relative crystallinity of various banana starch varieties are presented in Table 2. The RC of banana starch is 23.54–56.92% with A-, B-, or C-crystalline type. The RC of banana flour ranged from 31.9% to 36.6% while plantain starch has RC ranging from 18.4% to 21.9%.

### 3.3. Pasting Properties

The pasting properties of starch are analyzed using a Rapid Visco Analyzer (RVA) to provide information on the alteration of starch paste viscosity produced by heating and cooling starch in water. Pasting properties include pasting temperature (PT), peak viscosity (PV), holding viscosity (HV), breakdown viscosity (BV), final viscosity (FV), and setback viscosity (SV).

**Figure 2:**
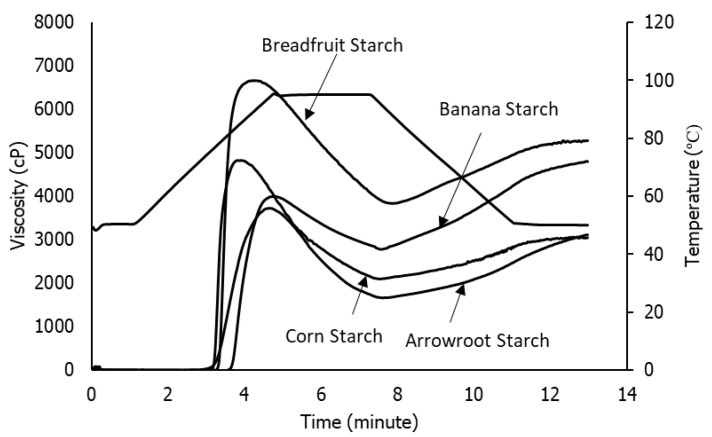
Viscoamylograph of native banana, breadfruit, corn, and arrowroot starches.

The botanical source of starch is one of the factors that affects the pasting properties of starch (Figure 2). Banana starch has a lower peak viscosity than breadfruit and corn starch. Banana starch also has the lowest breakdown viscosity, which indicates that it has the highest thermal stability among others.

The pasting properties of different banana starch cultivars have been reported [19,20,21,22,28]. Marta et al. [20] found that the cultivar affects the pasting properties of banana starch, i.e., PT, PV, BV, and SV of banana starch. The cooking banana has higher PT, PV, HV, FV, and SV, but lower BV compared to the plantain starch. Starch with higher PV and FV is more suitable as a thickener for food products that require high-temperature processing such as sterilization. The lower BV of banana starch indicated that the starch was more stable and resistant to a mechanical and heating process. Banana, which is cultivated and grown in Brazil, has a PT of 74.41–79.85 °C, PV 371.71–483.35 RVU, and BV 106.77–269.75 RVU [41]. Pelissari et al. [16] have reported that pasting properties of flour and starch extracted from green banana demonstrated a significant difference, banana starch having a lower PT, PV, BV, and FV than banana flour. The presence of other components and the reaction between amylose and the other components affect the pasting properties of banana starch [20].

Furthermore, Alimi et al. [42] reported that the plantain banana starch needs more thermal energy to form paste than the cooking banana starch (PT is 83.15 °C and 75.10 °C, respectively). The lower PV indicated the stronger cohesive strength between the starch granules, which causes a greater amount of heat energy needed to swell the starch. Plantain starch has a lower PV compared to cooking banana starch. The pasting properties of banana starch from various varieties are portrayed in Table 3.

### 3.4. Thermal Properties

Thermal properties of starch-based systems provide information about the gelatinization process, which occurs when the molecular order of starch granules is permanently lost and starch granules melt to generate the gel structure. The analysis of the thermal properties of starch is based on the heat absorption or loss that occurs as a result of phase changes (such as melting or crystallization) or chemical processes (for example, chemical decomposition) when a system is heated [45]. Several techniques may be used to analyze the thermal properties of starch. The commonly used technique to analyze the thermal properties of starch is using Differential Scanning Calorimetry (DSC), which measures the energy absorbed by the starch system as a function of temperature. DSC provides several thermal parameters by analyzing the endothermal curve, such as onset temperature (*T_o_*), peak temperature (*T_p_*), conclusion temperature (*T_c_*), thermal transition temperature (*T_c_* − *T_o_*), and the overall enthalpy (ΔH).

The thermal properties of banana starch are varied depending on the cultivar and other factors. Table 4 portrays that the *T_o_*s of cooking and plantain banana starch were 59.98–76.02 °C and 76.02 °C, respectively. The *T_o_* of banana starch is slightly higher than the *T_o_* of Mung bean starch [46], potato starch [47], sweet potato starch [48], rice starch [49], and wheat starch [50].

Furthermore, Agama-Acevedo et al. [44] reported that the plantain banana has higher *T_o_*, *T_p_*, *T_c_*, and *T_c_* − *T_o_* compared to the cooking banana planted in Mexico. The thermal properties analysis of the starch system using DSC also provides information about the gelatinization enthalpy or ΔH, measured as Joule per gram of sample. The internal arrangement of starch granules was measured by gelatinization enthalpy. A lower ΔH value indicates poor starch molecular structure. The ΔH value is related to the changes that occur during the melting of the crystallites and serves as a measure of the degree of crystallinity or damage in the starch structure before gelatinization. The ΔH value of banana starch ranged from 9.45 to 16.68 J/g. ΔH value is inversely related to amylose concentration, tiny granules, and disordered starch structure [51].

### 3.5. Functional Properties

Functional properties of starch include several parameters, i.e., swelling power (SP), solubility (SOL), water absorption capacity (WAC), oil absorption capacity (OAC), and freeze-thaw stability (FTS). The swelling power and solubility are useful metrics for determining the starch granule’s integrity. The proportion of soluble particles in the dry sample determines the solubility, allowing treatments based on gelatinization, dextrinization, and subsequent starch solubilization to be used [52]. The functional properties of banana starch from various varieties are portrayed in Table 5.

The swelling power and solubility of banana starch were 1.38–97.96 g/g and 1.29–11.76%, respectively. Banana starch also has relatively low freeze-thaw stability, indicated by the high syneresis of 12.16–44.40%. The WAC banana starch has a range from 130.45% to 251%. Meanwhile, plantain starch has a lower range of WAC value compared to cooking banana starch, which is 54.40% to 65.40%. The oil absorption capacity of banana starch ranges from 136.77% to 194.05%.

### 3.6. Digestibility

Digestion of starch granules is a complex process, which includes various phases: the diffusion of enzymes towards the substrate on the impact of the porosity of the substrate, enzyme adsorption on starch-containing materials, and hydrolytic events. Starch hydrolysis is also influenced by the crystallinity and granule surface properties of the native starch.

Increased blood glucose concentration after consuming foods containing high starch is due to the process of amylolysis of starch in the gastrointestinal tract, particularly maltose, maltotriose, and α-limit dextrin. Based on the length of time starch is digested in human digestion, starch can be classified into rapidly digestible starch, slowly digestible starch, and resistant starch [54]. Rapidly digestible starch causes an increase in blood glucose levels immediately after consumption, while slowly digestible starch will be fully digested in the small intestine at a slower rate than rapidly digestible starch. Resistant starch is not further digested in the small intestine but can be fermented in the large intestine into short-chain fatty acids [55].

Some of the digestibility features of native banana starch, which usually expressed as rapidly digested starch (RDS), slowly digested starch (SDS), and a resistant starch fraction (Table 6). RDS and SDS are considered available starch, which means the starch fraction that can be digested by digestion enzyme in the human’s small intestine, while RS is considered the starch fraction that cannot be digested by the enzyme that is produced by the human small intestine.

Native banana starches exhibit a low digestibility feature with RS content ranging from 65.3% to 98.98% (Table 6), which is much higher if compared to starches from wheat [56], pearl millet [57], and rice starch [58]. Furthermore, Toutounji et al. [59] reported that the digestibility of native starches, aside from their crystallinity, could also be affected by their granule morphology and their granule surface characteristics (e.g., the smoothness of the granule surface).

Marta et al. [8] reported that the banana starch has a smooth surface, i.e., no cracks or pores on its granule surface. This feature of granule surfaces could explain their resistance to the digestive enzyme. It would cause the digestion mechanism called “exo-pitting” which means the enzyme will digest the starch from the outside since the surface is impermeable to amylases [62]. The naturally occurring pores on the surface of the starch granule could increase the effective surface area to form greater enzyme-substrate complexes, causing a faster rate of enzymatic digestion of the starch.

## 4. Modification Practices

The properties of starch can be modified through physical, chemical, and enzymatic methods. Starch can be physically modified to change particle size and improve water solubility. The physical methods involve the treatment of native starch granules under different moisture and temperature combinations, shear pressure, and irradiation to alter the physical size of starch granules [12]. The physical method is the better method because it does not involve any chemical treatment.

### 4.1. Physical Method

Physical alterations of starch are changes given by physical treatments that do not result in modification of the starch polymer molecules’ D-glucopyranosyl units. By modifying the starch using physical treatment, only the arrangements of the starch polymer within the granules and the overall starch structure are changed. These changes could provide a significant effect on the properties of the starch, such as the properties of the pastes, gels, and the digestibility properties of the modified starch. Many modified starch users are interested in physically modified starch because it does not require any chemical reagent, thus they do not need to label the starch as a modified starch product. Physical modification of starch is usually divided into thermal (heat-moisture treatment, annealing, pre-gelatinization, extrusion, etc.) and nonthermal modification (ultrasound, high-pressure treatment, pulsed electric field, etc.).

#### 4.1.1. Heat-Moisture Treatment

Heat-moisture treatment (HMT) of starch is a physical treatment in which starches are treated at varying moisture levels (<35% moisture *w*/*w*) during a certain period (15 min–16 h) at a certain temperature range (84–120 °C) [63,64]. Among the physical methods, HMT has several advantages such as being cost-effective, safe, and more suitable for commercial use [65].

HMT promotes the interaction of polymer chains by disrupting the crystalline structure and dissociating the double-helical structure in the amorphous region, followed by the rearrangement of the disrupted crystals [64]. Heat-moisture treatment can improve the properties of native starch, such as reducing the swelling volume and solubility and increasing the thermal stability of native banana starch. HMT may also increase the slowly digestible starch content of native banana starch [8,29], so it can be recommended as a functional food for diabetic patients. Food products with high slowly digestible starch content have a relatively slow effect on increasing the Glycemic Index (GI). Clinical data suggest that a diet with low IG is associated with a reduced risk of diabetes and cardiovascular disease [66].

#### 4.1.2. Annealing

Annealing (ANN) is another starch modification technique which heats the starch and holds it in excess water (>40%) for a certain amount of time. The starch is heated at the temperature above its glass transition temperature (*T_g_*) and below onset temperature of gelatinization (*T_o_*). ANN alters the physicochemical characteristics of starch by enhancing its crystalline perfection and promoting interactions between starch chains. The ANN process retains the intact granular form of the starch with some notable changes on the surface of the starch granule, including crack and pore formation and increased roughness [67].

Some previous studies have reported the ANN modification on banana starch [29,68]. De la Rosa Millán et al. [68] reported that after annealing banana flour, some of the starch granular shapes retain their elongated lenticular shape, but some of them are swollen. The birefringence of the starch, however, is still retained after the ANN modification. Annealing-treated starch has lower amylose leaching, RDS, and SDS values but a higher RS value and *T_o_*, *T_p_*, and *T_c_* values compared to its native counterpart. Furthermore, Cahyana et al. [29] reported that there was not a noticeable change in the starch granule morphology after annealing, which was indicated by the absence of aggregation, cohesive structure, holes, or fissures in the annealing-treated granules. The pasting temperature, peak, and breakdown viscosity of annealing treated starch were significantly higher than the native form. The annealing modified flour has a lower digestible starch content than the native form [68], which is not aligned with another study [29].

#### 4.1.3. Pregelatinization

Pregelatinized starch, often known as instant starch, is modified starch which can be redissolved easily in room temperature water and increase viscosity without any heat treatment. Pregelatinized starch (PGS) has been gelatinized and pasted before it was dried and finally ground. These starches’ granules are often substantially degraded, and they are normally soluble in room-temperature water. Due to the modification process, the newer product, which is still intact but has a gelatinized starch granule, is formed. The starch granules have lost crystallinity and swell to form a paste when it is incorporated into the water.

Several studies have reported the application of pregelatinized banana starch and flour in food products. Loypimai and Moongngarm [69] have reported that the addition of pregelatinized banana flour significantly improves the rehydration rate of the instant porridge. The addition of pregelatinized banana flour also improved the dietary fiber content, RS content, and antioxidant capacity of the instant porridge. Furthermore, Olatunde et al. [28] reported that the water-absorbing capacity (WAC) of starches is significantly increased after subjecting banana and plantain starch to the pre-gelatinization modification. The WAC of native banana and plantain starch was 136.53% and 130.45%, respectively. After pre-gelatinization, the WAC of both native cooking banana and plantain starches increased to 155.47% and 203.25%, respectively. Furthermore, Azaripour and Abbasi [70] also reported that the pregelatinized cornstarch has significantly higher WAC than the native one. The increase in the water absorption index of native starch after pre-gelatinization was also reported on corn starch [71], rice starch [72], and maize starch [73].

### 4.2. Chemical Method

Chemical modification is accomplished by inserting a functional group into the polymer molecule of the starch granule in its native form, resulting in different changes in the physicochemical characteristics of the starch molecule. Chemical modification entails changing the physiochemical characteristics of starch by adding new chemical or functional groups into the starch without causing physical changes to the shape and size of the molecule [74]. Starch modification by oxidation, etherification, esterification, cationization, and cross-linking are the most modifications used for starch.

#### 4.2.1. Esterification

The starch esterification process could produce acetylated starch, monostarch phosphate, and starch 2-octenyl succinate. Acetylated starch esters are usually made by subjecting the starch granules to the alkaline slurry and acetylating them using acetic anhydride where the acetyl group will replace the hydroxyl groups on C2, C3, and C6 positions. The resulting starch, when it has a degree of substitution (DS) of 0.09 or lower (which is the maximum DS allowed in acetylated starch for foods), generally has lower gelatinization and pasting temperature, has better clarity after the pasting process, and better stability to retrogradation and freeze-thaw process when compared to the native/uncooked starch [75]. Acetylated starch also has a higher swelling capacity and peak viscosity but lower final paste viscosity than the native starch [74].

The acetylation process promotes cracks on the starch granule surface. The acetylation increases swelling capacity, water absorption capacity, and pasting properties, and decreases the solubility of native banana starch [76]. Conversely, Olatunde et al. [28] have reported that the pasting properties parameters of acetylated banana starch were lower than the native form except for the breakdown viscosity. Furthermore, Olatunde et al. [28] and Dumancela et al. [77] reported an increase in swelling capacity and solubility of acetylated banana starch. The increase in swelling power and solubility of acetylated starch were also reported on sorghum [78], yam [79], and low DS cassava starch [80].

Banana starch can also modify using octenyl succinic anhydride by esterification. Carlos-Amaya et al. [81] have reported that the starch with 0.016 degrees of substitution (DS) was obtained by adding 0.60 g of 2-octen-1ylsuccinic anhydride for 100 g of banana starch. The study indicated that the OSA-modified banana starch has a significantly higher degree of crystallinity compared to the native one. It might be due to starch molecules in the amorphous lamella and a portion of the crystalline lamella being destroyed during the reaction. The increased crystallinity can be correlated to a higher number of short chains generated during the chemical modification, as observed in oxidized starch. The OSA modification could also significantly increase the RDS and SDS while decreasing the RS content of native banana starch. OSA banana starch has a higher peak viscosity, but lower *T_o_*, *T_p_*, *T_c_*, and ΔH compared to the native starch [82]. The decreases in *T_o_*, *T_p_*, *T_c_*, and ΔH after OSA modification are also reported on wheat starch, rice starch, corn starch, waxy corn starch, kidney bean starch, and sweet potato starch [83]. Furthermore, Bajaj et al. [83] have explained that the decrease in transition temperatures, particularly *T_p_*, might be due to the insertion of octenyl-succinic groups into highly stabilized starches, resulting in chain instability and linearity. Bello-Pérez et al. [84] have reported that OSA modification to banana starch improves the emulsion stability of native banana starch. The improvement in emulsion stability was also supported by the change in the sample’s viscoelastic properties. The addition of OSA-modified banana starch to the emulsion made the G′ higher than G″ (it was G″ > G′ when native banana starch was used). The previous study reported the application of OSA-modified taro starch as an emulsion stability improvement in food products [85].

#### 4.2.2. Etherification

Starch etherification is one of the starch stabilization processes. In most cases, native starch was etherified by reacting it with alkyl halides, acrylonitrile, or alkylene oxides in the presence of an alkaline catalyst. The active area in starch where etherification occurs was determined by the botanical source, the alignment of amylose and amylopectin, and the distribution of amorphous and crystalline regions [86]. Hydroxypropyl starch was created by reacting starch granules with propylene oxide in an alkaline slurry to create modest amounts of etherification. The introduction of hydrophilic hydroxypropyl groups on starch chains disrupts the starch granule structure and weakens inter- and intramolecular hydrogen bonding. As a result, increasing the accessibility of starch granules to water changes starch gelatinization and retrogradation behavior. The hydroxypropylated starch has lower pasting temperatures, so it is easier to cook and produces a clear paste that does not retrograde and has better freezing-thawing stability [75].

Waliszewski et al. [35] have reported that the hydroxypropylated banana starches have a higher water-binding capacity, swelling power, and solubility, but lower pasting temperature and paste clarity compared to the native form. Lee et al. [87] reported that the quantity of propylene oxide used in hydroxypropylation has the most pronounced effect on the Saba banana starch properties compared to pH and temperature reaction. The hydroxypropylated Saba banana starch was easier to cook, had higher freeze-thaw stability, more resistance to retrograde, and lower pasting properties and temperature. The decreasing *T_o_*, *T_p_*, *T_c_* and ΔH values after hydroxypropylation were also reported on hydroxypropylated finger millet [88], *Dioscorea alata* starch [89], high-amylose corn starch [90], and yellow sorghum starch [91].

Hydroxypropylated banana starch has a higher granule swelling capacity and solubility compared to the native banana starch. The increase in granule swelling and solubility was also reported by several previous studies [92,93,94,95,96]. The increasing granular swelling and solubility of the hydroxypropylated starch might be due to the starch granules becoming more hydrophilic because of the incorporation of the hydroxypropyl groups.

Hydroxypropylated banana starch has a lower tendency toward retrograde and syneresis. The gel clarity of the hydroxypropylated banana starch was better than the native one. Amylose crystallization, intermolecular associations (including possible amylose–amylopectin interactions), and crystallization of amylopectin molecules’ exterior chains are reduced by the conversion of hydroxyl groups of starch molecules into larger hydroxypropyl groups via the reduction in inter-and intramolecular forces. The higher the degree of molar substitution (MS), the higher the gel clarity of the hydroxypropylated starch [97].

#### 4.2.3. Cross-Linking

Cross-linking occurs in the majority of modified food starches. When starch granules are treated with bifunctional reagents that react with hydroxyl groups on two distinct molecules or neighboring chains inside the granule, cross-linking occurs. The most common method of cross-linking is the formation of distarch phosphate esters. The main food-grade reagents are sodium trimetaphosphate (STMP), monosodium phosphate (SOP), sodium tripolyphosphate (STPP), epichlorohydrin (EPI), phosphoryl chloride (POCl_3_), a mixture of adipic acid and acetic anhydride, and vinyl chloride [74].

Carlos-Amaya et al. [81] studied the effect of double modification (cross-linking and esterification) using 0.05% epichlorohydrin (EPI) on the physicochemical and digestibility properties of banana starch. The study found that cross-linked banana starches have a higher crystallinity but a lower peak viscosity than the native/uncooked banana starch, which indicates that cross-linking of banana starch using 0.05% EPI could hinder the swelling of the starch at the holding period of RVA (95 °C for approximately 5 min). Furthermore, Orsuwan and Sothornvit [98] reported that the cross-linked banana starch has significantly decreased the swelling power and solubility of its native counterpart.

#### 4.2.4. Oxidation

Starch oxidation is a frequently used alternative technique for enhancing starch characteristics. Commercially, oxidized starch is made by reacting starch with a particular amount of oxidant at a regulated pH and temperature. When starch is exposed to higher concentrations of an oxidizing agent (oxidant), the outcome is oxidized starch. To oxidize starch, oxidants such as potassium permanganate, sodium hypochlorite, hydrogen peroxide, peracetic acid, nitrogen dioxide, and chromic acid have been used. The most often used commercial oxidant for this method is sodium hypochlorite [75].

Sánchez-Rivera et al. [99] reported that the higher the active chlorine concentration used, the whiter the color of the starch. The oxidation also increases the peak viscosity of banana starch, which was caused by the introduction of the carbonyl and carboxyl groups to the starch molecule, facilitating the granules to swell [99].

The oxidation decreased the crystallinity of native banana flour and did not change the starch crystalline type [100]. This result was aligned with another study on oxidized jackfruit seed starch [101]. Oxidation treatments decreased WHC, solubility, and whiteness index while increasing the swelling volume, freeze-thaw stability, and gel strength of banana flour [100]. The increasing swelling power after the oxidation process also occurred on oxidized jackfruit seed starch using 4–5% sodium hypochlorite [101] and oxidized whole grain flour using ozone [102]. On a contrary, Halal et al. [103] reported a decreasing swelling power and increasing solubility of the oxidized barley starch. Cahyana et al. [100] reported the reduction of pasting temperature, breakdown and setback viscosity, and increased peak viscosity after oxidation treatment, which is in agreement with other studies on barley starch [103] and oxidized jackfruit seed [101].

## 5. Potential Application in Food Products

Food processing is an attempt to transform the shape of food, including agricultural and animal crops, into the intended food items so that the food has additional value in terms of quality, acceptance, and storage stability. Banana is a highly perishable fruit, so needs to be processed to improve self-life such as processing into flour or starch. Transforming a banana into flour or starch not only improves its self-life but is also widely used in many different products. Banana flour can apply as a wheat flour substitute in pasta and bakery products (pasta, cake, bread, cookies, and biscuits) and banana starch can apply as a thickening agent, fat replacer, edible coating, etc.

Banana starch has not been used commercially. Native banana starch has different characteristics depending on some factors such as the variety and growing environment. Ambon variety banana starch has good freeze-thaw stability [20], so it is suitable for application in frozen food products. Several studies found that native banana starch has high levels of resistant starch 65–99% [7,8,20,39,60]. Resistant starch is a fraction of starch that cannot be digested by digestive enzymes, so it does not increase blood glucose levels when consuming it. Therefore, banana starch is recommended as a functional food product for diabetic patients.

Native banana starch was modified to improve its application. HMT starch can increase the thermostability of banana starch and decrease swelling volume and solubility [8,29,104,105,106]. Based on its pasting profile and functional properties, HMT starch in flour can apply as a raw material for noodle making, thus producing the desired characteristics of noodles, such as not being easy to break, limited swelling, and low cooking loss [107]. Furthermore, HMT can increase the slowly digestible starch (SDS) of native banana starch [8,29]. Food with high SDS can be beneficial for maintaining satiety and physical performance of the body, in addition to lowering blood fat levels and insulin resistance associated with glucose homeostasis [108].

OSA-modified starch also has the potential to be used as a fat replacer. Some previous studies have reported OSA-modified starch to have amphiphilic properties [83,84,109], making it possible to dissolve in polar and nonpolar systems. OSA-modified starch can be applied as an emulsifier, fat replacer, and encapsulation agent. As a fat replacer, OSA-modified starch can replace fat in fat-based products such as mayonnaise, salad dressing, margarine, etc., resulting in low fat-based products.

Several previous studies have reported the application of banana starch (in flour form) in food products such as pasta [110,111], bread [112], cookies [113], and biscuits [114]. In these products, banana flour is used to replace wheat flour, reducing the gluten content in the product. Pasta made from green banana flour was digested more slowly than wheat pasta [110,111]. In line with pasta, bread made from banana flour also has lower digestibility than wheat bread. The banana flour bread had a lower hydrolysis index (HI) and projected glycemic index (pGI). Substitution of 30% wheat flour with banana flour did not affect the aroma, flavor, or general acceptance of steam white bread, indicating banana flour bread is a highly marketable bread with enhanced functional value [112]. Aparicio-Saguilán et al. [113] reported cookies made from substitution wheat flour with autoclave-treated lintnerized banana starch to have a low digestibility and moderate GI. Furthermore, Cahyana et al. [114] found that biscuits made from the substitution of wheat flour with heat-treated banana flour have a high slowly digestible starch and have the potential to be a long-lasting energy product.

Banana starch can also be used as the base ingredient in a biodegradable film. However, the film produced from banana starch still has several flaws in its physical properties. Therefore, some research groups attempted to reinforce the banana starch film by adding another compound to improve the film’s physical properties. Pelissari et al. [115] reported that adding cellulose nanofibers extracted from banana peel to the banana starch film significantly enhanced its tensile strength, Young’s modulus, opacity, and crystallinity, and made the film more resistant to water. Furthermore, Viana et al. [116] reported that the film made from heat-moisture-treated banana starch has a more cohesive matrix with better mechanical properties than the film made from native banana starch. The enhanced mechanical properties of the film from heat-moisture-treated banana starch are related to the lower pasting viscosity and retrogradation. The heat-moisture-treated banana starch film also has lower water vapor permeability.

## 6. Potential Application in Non-Food Products

Banana starch can be utilized not only on food products but also for non-food products. Banana starch is one of the potential primary materials to produce a bio-based film, such as film-type packaging [117]. Furthermore, banana starch can be utilized as the primary material to produce bio-based packaging films [118,119,120,121]. However, because starch, including banana starch, imparts poor flexibility and poor barrier properties to both water and air, some chemical addition and modification practices are often applied [117]. To improve the barrier properties of banana starch, Pongsuwan et al. [122] utilize the banana inflorescence waste, which contains a high amount of fiber, as a filler for starch-based bioplastics which gave the bioplastics better physical properties, water, and thermal resistance. The addition of filler or banana starch modification in production, banana starch-based bio film was also reported by several studies [123,124,125]. Starch’s ability to absorb water easily often causes it to lose some of its dimensional stability and mechanical qualities [126]. Some materials, however, can be incorporated to improve this physical barrier properties of starch-based film. Cellulose can be used to improve the physical barrier properties of starch-based film. Azmin et al. [127] reported that by incorporating bagasse fiber into a bioplastic film made from cocoa pod husk cellulose, the water-vapor transmission rate decreased. More recently, Oyeoka et al. [128] studied the incorporation of hyacinth water cellulose nanocrystal to a nanocomposite film made from polyvinyl alcohol (PVA)/gelatin composite and reported that by incorporating water hyacinth cellulose nanocrystal, the water vapor permeability and the moisture uptake of the PVA/gelatin composite film was decreased, indicating an improvement water barrier properties of the bio-based film. Furthermore, the application of cellulose nanocrystal and cellulose nanofiber to improve barrier properties of starch-based film was also reported by Zhang et al. [129]. They reported that incorporating cellulose nanocrystal could improve the mechanical strength of the starch film and incorporating cellulose nanofiber imparts an improvement on thermal stability of the starch-based film. These previous studies on improving bio-based films mechanical properties using cellulosic material could be further applied to the starch film made using banana starch in order to reinforce the barrier properties of the banana starch-based film.

Starch, other than being utilized as the biofilm-making material, also has the potential to be transformed as porous carbon materials to produce an environment-friendly capacitor with high efficiency. Supercapacitors are being evaluated as prospective energy storage devices in applications with rising power needs because of their superior electrochemical characteristics [130]. From the author’s perspective, banana starch could also be utilized as the primary material to produce electrode materials or gel electrolytes for supercapacitors. This statement was based on the research done by Kasturi et al. [131] who utilized *Artocarpus heterophyllus* seed starch as a porous material for capacitor and flexible film from *Manihot esculenta*. They reported that this all-solid-state electric double-layer capacitor has great sustainability with superior electrochemical performance and stability. Furthermore, Kumar et al. [132] reported that cornstarch could be utilized as a porous carbon material by the carbonation process. According to the previous research, banana starch had the potential to be utilized as the precursor to synthesis porous carbon materials to produce eco-friendly capacitors with high efficiency in conducting electricity.

## 7. Conclusions and Future Research

Banana starch has good potential to be developed as an ingredient in the food industry. Banana starch has a high level of resistant starch, so it can be used as an ingredient in functional foods, such as food for diabetics. In addition, several banana varieties also have unique functional properties, such as having good stability when stored at low and frozen temperatures (high freeze-thaw stability), making them suitable for use as ingredients in frozen foods.

However, native banana starch has some drawbacks, so modifications are needed to expand its application. Several studies on the modification of banana starch, both physically and chemically, indicated that the modification treatment could improve the characteristics of native banana starch, such as increased thermal stability, amphiphilic characteristics, increasing slowly digestible starch (SDS) content, and others. Food products with high SDS content are also suitable for consumption by diabetic patients. Furthermore, SDS can be used as an ingredient for long-lasting energy products, because products high in SDS have a slower rate of digestion, decrease glucose and insulin response, and increase the subsequent feeling of satiety.

Further studies are needed to modify banana starch using other methods, such as enzymatic and nonthermal physical (ultrasonication, pulse electric field, high-pressure treatment, etc.), to obtain more information and alternative methods to improve banana starch characteristics. Conversely, the application of banana starch (in the form of flour) as a substitute for wheat flour must be studied, not only partially replacing but also 100% replacing the use of wheat flour to produce gluten-free products as a functional food for people with special diets, i.e., celiac disease and autism.

## Figures and Tables

**Table 1:** Chemical composition of starches from various banana varieties.

Variety	Moisture (%)	Ash (%)	Lipid (%)	Protein (%)	Total Starch (%)	Fiber (%)	Amylose (%)	Reference
*Musa* AAB—Mysore Var.	12.30	0.09	NR	0.44	90.08	NR	37.88	[21]
White Manzano	NR	0.08	0.06	0.22	99.6	NR	30.3	[22]
Dwarf Cavendish	NR	0.09	0.09	0.34	99.5	NR	26.5	
Nanicao	14.00	0.13	0.18	0.61	84.94	0.14	NR	[19]
Grand Naine	10.82	0.09	0.26	0.38	87.86	0.59	NR	
Maca	8.60	0.26	0.36	0.63	89.67	0.47	NR	
Prata-Ana	9.90	0.38	0.46	0.99	87.88	0.38	NR	
Fhia 18	10.28	0.34	0.01	1.09	88.08	0.18	NR	
Terra var.	7.38	0.11	1.82	1.77	NR	NR	33.11	[23]
Banana (Kluai Khai var)	7.83	0.05	0.03	0.17	NR	NR	20.32	[15]
Banana (Hom Tong var.)	7.30	0.06	0.10	0.21	NR	NR	13.36	
Banana (Namwa var.)	7.16	0.05	0.12	0.20	NR	NR	28.03	
Banana (Enano var.)	7.03	1.43	0.73	0.92	NR	NR	25.38	
Banana (Morado var.)	8.75	1.17	0.73	0.83	NR	NR	21.99	[24]
Banana (Valery var.)	8.96	1.27	0.78	0.93	NR	NR	19.32	
Banana (macho var.)	7.72	1.11	0.82	0.98	NR	NR	26.35	
Banana (Terra var.)	8.00	0.03	0.02	0.97	94.80	0.28	35.00	[16]
Banana (Karpuravali var.)	10.55	1.24	0.22	1.17	87.30	NR	27.66	[25]
Banana (Poovan var.)	9.53	0.78	0.25	0.67	81.71	NR	23.10	
Banana (Sevvazhai var.)	11.18	1.68	0.23	1.15	89.62	NR	32.05	
Banana (Thenvazhai var.)	8.76	2.08	0.28	0.86	85.63	NR	24.63	
Plantain (Fench horn var.)	11.56	0.05	NR	NR	NR	NR	29.96	[26]
Plantain (Cadaba var.)	12.86	0.29	NR	NR	NR	NR	30.66	
Plantain (Agbaba var)	13.15	0.45	NR	NR	NR	NR	30.91	
Banana (Macho var.)	9.90	0.54	2.46	2.03	98.10	NR	NR	[27]
Banana (Honduras var.)	13.60	0.92	0.72	2.16	69.39	0.47	42.07	[28]
Plantain (Agbaba var)	11.20	0.62	0.44	2.53	63.90	0.72	38.79	
Banana (Kapas var.)	8.92	NR	0.24	0.96	90.02	NR	38.63	[20]
Banana (Kepok var.)	6.83	NR	0.06	1.01	82.69	NR	40.88	
Banana (Ambon var.)	7.98	NR	0.05	1.25	81.64	NR	32.56	
Banana (Nangka var.)	7.97	NR	0.12	1.58	81.53	NR	37.78	
Plantain (Gros Michel var.)	7.8	0.50	0.8	1.1	74.9	NR	22.76	[18]
Plantain (Dominico Hartón var.)	8.2	0.34	0.5	0.9	84.0	NR	31.12	
Plantain (FHIA 20 var.)	7.3	0.29	0.6	0.9	83.5	NR	28.58	

NR: Not Reported

**Table 2:** The crystalline type and relative crystallinity of starch form various banana varieties.

Variety	Relative Crystallinity (%)	Crystalline Type	Reference
Banana (Kapas var.)	37.78	B-type	[8]
Banana (Nendran var.)	35.96	A-type	[37]
Banana (Kapas var.)	35.8	B-type	[34]
Banana (Hom Khieo var.)	23.54	B-type	[38]
Banana (Namwa var.)	26.84	B-type	
Banana	30	B-type	[24]
Plantain (Dominico Harton var.)	21.9	C-type	[18]
Plantain (FHIA 20 var.)	21.4	C-type	
Plantain (Gros Michel var.)	18.4	C-type	
Banana (Musa Dwarf Red banana var.)	29.84	C-type	[39]
Banana (Pisang Awak var.)	33.02	C-type	
Banana (Cavendish)	31.26	C-type	
Banana (Musa Cocinea var.)	32.84	C-type	[40]
Banana (Williams banana var.)	33.02	C-type	
Banana (Nangka var.)	33.2	B-type	[20]
Banana (Ambon var.)	34.76	B-type	
Banana (Kepok var.)	39.36	B-type	
Banana (Kapas var.)	38.64	B-type	

**Table 3:** The pasting properties of banana starch from various varieties.

Variety	PT (°C)	PV (cP)	HV (cP)	BD (cP)	FV (cP)	SV (cP)	Reference
Kapas	79.11	4037	3049	988	5308	2258	[20]
Kepok	80.70	3996.5	2778	1218.5	4796	2018	
Ambon	77.27	4529	3294.5	1234.5	4506.5	1212.25	
Nangka	76.58	4535.5	3409.5	1126	4995.5	1586	
Culinary Banana	81.80	4507	4060	447	4214	154	[43]
Cooking Banana starch	75.10	6403	5443	960	7090	1647	[42]
Plantain banana starch	83.15	5447	4783	664	7743	2960	
Mysore	79.1	5455.3	3034	2421.3	3985	951	[21]
Macho	81.2	4152	NR	1290	4189	1327	[44]
Enano	77.2	4026	NR	1415	4072	1461	
Morado	70.5	4141	NR	1648	3412	919	
Valery	76.6	4848	NR	1534	3986	672	

NR: Not Reported

**Table 4:** Thermal properties of banana starch from various varieties.

Variety	T_o_ (°C)	T_p_ (°C)	T_c_ (°C)	T_c_–T_o_	ΔH (J/g)	Reference
Macho	69.46	74.4	81.6	NR	13.0	[36]
Criollo	71.4	75.0	80.4	NR	14.8	
Nanicão	68.07	70.58	73.73	5.66	14.73	[19]
Grand Naine	68.65	71.11	74.21	5.56	13.22	
Macã	69.23	72.17	75.36	6.13	10.61	
Prata-Anã	67.79	71.02	74.64	6.85	12.94	
FHIA 18	68.5	71.69	74.67	6.17	9.45	
Mysore	67.54	75.18	87.56	20.02	12.38	[21]
Macho	74.6	78.7	92.4	NR	15.1	[24]
Valery	71.9	76.2	88.71	NR	14.8	
Morado	60.9	70.2	83.2	NR	10.4	
Enano	71.3	78.4	91.5	NR	14.9	
Valery	72.31	75.90	90.19	17.88	12.86	[44]
Morado	59.89	68.03	81.25	21.36	10.65	
Enano	71.30	77.21	91.56	20.26	14.56	
Macho (plantain)	76.02	79.98	99.08	23.06	14.53	
Terra starch (plantain)	72.1	74.9	78.3	6.3	14.7	[16]
Terra flour (plantain)	72.3	76.2	80.5	8.2	13.0	
Karpuravali	63.70	71.12	84.34	NR	16.05	[25]
Poovan	62.08	69.63	82.35	NR	15.06	
Sevvazhai	65.54	72.81	87.99	NR	16.68	
Thenvazhai	63.14	70.31	84.63	NR	16.41	

NR: Not Reported

**Table 5:** Functional properties of banana starch from various varieties.

Variety	SP (g/g)	Sol (%)	Syneresis (%)	WAC (%)	OAC (%)	Ref
Nanicao	13.20	5.44	NR	NR	NR	[19]
Grand Naine	15.19	6.85	NR	NR	NR	
Maca	14.42	9.88	NR	NR	NR	
Prata-Ana	14.66	11.61	NR	NR	NR	
Fhia 18	14.88	6.67	NR	NR	NR	
Banana (Honduras Var.)	1.38	NR	NR	136.53	136.77	[28]
Plantain (Agbaba var)	1.99	NR	NR	130.45	194.05	
Banana (Monthan Var.)	NR	NR	NR	176.10	186.56	[23]
Banana (Terra var.)	24.00	13.7	NR	NR	NR	[16]
Plantain (Fench horn var.)	9.48	7.49	NR	65.50	NR	[26]
Plantain (Cadaba var.)	10.76	3.55	NR	54.40	NR	
Plantain (Agbaba var)	10.10	5.02	NR	62.90	NR	
Banana (Musa AAA Cavendish)	85.13	5.31	NR	NR	NR	[53]
Banana (Musa ABB Bluggoe)	89.64	1.29	NR	NR	NR	
Banana (Musa ABB Pisang Awak)	97.96	9.70	NR	NR	NR	
Banana (Musa AA Pisang Mas)	82.45	8.69	NR	NR	NR	
Plantain (Kapas Var.)	NR	NR	12.16	156.00	NR	[9]
Plantain (Kapas Var.)	NR	4.63	26.93	137.00	NR	[34]
Plantain (Kapas var.)	NR	4.48	22.23	149.00	NR	[20]
Plantain (Kepok var.)	NR	4.42	32.14	151.00	NR	
Plantain (Nangka var)	NR	4.36	17.62	174.00	NR	
Banana (Ambon var)	NR	3.21	0.59	178.00	NR	

NR: Not Reported

**Table 6:** Digestibility properties of banana starch.

Variety	RDS (%)	SDS (%)	RS (%)	Reference
Musa Dwarf Red banana	2.34	12.12	85.54	[39]
Musa ABB Pisang Awak	3.03	4.81	92.16	
Musa AAA Cavendish	8.19	6.61	85.20	
Green Banana	1.7	4.3	94.1	[60]
Kapas	0.43	0.59	98.98	[8]
Musa coccinea	7.3053	14.7230	77.9718	[40]
Williams banana	8.3249	6.4137	85.2614	
Kapas	0.41	0.61	98.98	[9]
Gros Michel	4.31	11.82	83.87	[18]
Dominico Harton	1.24	8.73	90.03	
FHIA 20	3.19	5.10	91.71	
Macho	1.3	6.8	91.9	[7]
Enano	5.8	4.4	89.9	
Valery	7.2	4.7	88.1	
Morado	16.6	18.1	65.3	
Unripe Banana	3.2	13.2	65.8	[61]

## Data Availability

Not applicable.

## References

[1] Soorianathasundaram K., Narayana C.K., Paliyath G. (2016). Bananas and Plantains. Encyclopedia of Food and Health.

[2] Gao H., Huang S., Dong T., Yang Q., Yi G. (2016). Analysis of Resistant Starch Degradation in Postharvest Ripening of Two Banana Cultivars: Focus on Starch Structure and Amylases. Postharvest Biol. Technol..

[3] Gibert O., Dufour D., Giraldo A., Sanchez T., Reynes M., Pain J.P., Gonzalez A., Fernandez A., Diaz A. (2009). Differentiation between Cooking Bananas and Dessert Bananas. 1. Morphological and Compositional Characterization of Cultivated Colombian Musaceae (*Musa* sp.) in Relation to Consumer Preferences. J. Agric. Food Chem..

[4] Zhang P., Whistler R.L., BeMiller J.N., Hamaker B.R. (2005). Banana Starch: Production, Physicochemical Properties, and Digestibility—A Review. Carbohydr. Polym..

[5] Aurore G., Parfait B., Fahrasmane L. (2009). Bananas, Raw Materials for Making Processed Food Products. Trends Food Sci. Technol..

[6] Lii C.-Y., Chang S.-M., Young Y.-L. (1982). Investigation of the Physical and Chemical Properties of Banana Starches. J. Food Sci..

[7] Agama-Acevedo E., Nuñez-Santiago M.C., Alvarez-Ramirez J., Bello-Pérez L.A. (2015). Physicochemical, Digestibility and Structural Characteristics of Starch Isolated from Banana Cultivars. Carbohydr. Polym..

[8] Marta H., Cahyana Y., Djali M. (2021). Densely Packed-Matrices of Heat Moisture Treated-Starch Determine the Digestion Rate Constant as Revealed by Logarithm of Slope Plots. J. Food Sci. Technol..

[9] Marta H., Cahyana Y., Djali M. (2021). Pectin Interaction with Thermally Modified Starch Affects Physicochemical Properties and Digestibility of Starch as Revealed by Logarithm of Slop Plot. CYTA J. Food.

[10] Lovera M., Pérez E., Laurentin A. (2017). Digestibility of Starches Isolated from Stem and Root Tubers of Arracacha, Cassava, Cush-Cush Yam, Potato and Taro. Carbohydr. Polym..

[11] Jyothi A.N., Sajeev M.S., Sreekumar J.N. (2010). Hydrothermal Modifications of Tropical Tuber Starches. 1. Effect of Heat-Moisture Treatment on the Physicochemical, Rheological and Gelatinization Characteristics. Starch-Stärke.

[12] Xie S.X., Liu Q., Cui S.W. (2005). Starch Modification and Application.

[13] Taggart P., Mitchell J.R. (2009). Starch. Handbook of Hydrocolloids.

[14] Bello-Perez L.A., Agama-Acevedo E., Sayago-Ayerdi S.G., Moreno-Damian E., Figueroa J.D.C. (2000). Some Structural, Physicochemical and Functional Studies of Banana Starches from Two Varieties Growing in Guerrero, Mexico. Starch-Stärke.

[15] Nimsung P., Thongngam M., Naivikul O. (2007). Compositions, Morphological and Thermal Properties of Green Banana Flour and Starch. Agric. Nat. Resour..

[16] Pelissari F.M., Andrade-Mahecha M.M., Sobral P.J.d.A., Menegalli F.C. (2012). Isolation and Characterization of the Flour and Starch of Plantain Bananas (*Musa paradisiaca*). Starch-Stärke.

[17] Otegbayo B., Lana O., Ibitoye W. (2010). Isolation and Physicochemical Characterization of Starches Isolated from Plantain (*Musa paradisiaca*) and Cooking Banana (*Musa Sapientum*). J. Food Biochem..

[18] Chávez-Salazar A., Bello-Pérez L.A., Agama-Acevedo E., Castellanos-Galeano F.J., Álvarez-Barreto C.I., Pacheco-Vargas G. (2017). Isolation and Partial Characterization of Starch from Banana Cultivars Grown in Colombia. Int. J. Biol. Macromol..

[19] De Barros Mesquita C., Leonel M., Franco C.M.L., Leonel S., Garcia E.L., dos Santos T.P.R. (2016). Characterization of Banana Starches Obtained from Cultivars Grown in Brazil. Int. J. Biol. Macromol..

[20] Marta H., Cahyana Y., Djali M., Arcot J., Tensiska T. (2019). A Comparative Study on the Physicochemical and Pasting Properties of Starch and Flour from Different Banana (*Musa* spp.) Cultivars Grown in Indonesia. Int. J. Food Prop..

[21] Fontes S., Cavalcanti M., Candeia R., Almeida E. (2017). Characterization and Study of Functional Properties of Banana Starch Green Variety of Mysore (*Musa* AAB-Mysore). Food Sci. Technol..

[22] Hung P.V., Cham N.T.M., Truc P.T.T. (2013). Characterization of Vietnamese Banana Starch and Its Resistant Starch Improvement. Int. Food Res J..

[23] Parimalavalli R., Babu D. (2014). Comparative Study on Properties of Banana Flour, Starch and Autoclaved Starch. Trends Carbohydr. Res..

[24] Utrilla-Coello R.G., Rodríguez-Huezo M.E., Carrillo-Navas H., Hernández-Jaimes C., Vernon-Carter E.J., Alvarez-Ramirez J. (2014). In Vitro Digestibility, Physicochemical, Thermal and Rheological Properties of Banana Starches. Carbohydr. Polym..

[25] Chagam K.R., Haripriya S., Vidya P. (2015). Morphology, Physico-Chemical and Functional Characteristics of Starches from Different Banana Cultivars. J. Food Sci. Technol..

[26] Kiin-Kabari D., Sanipe P., Friday O. (2014). Physico-Chemical and Pasting Properties of Starch from Three Plantain Cultivars Grown in Nigeria. Afr. J. Food Sci..

[27] Núñez-Santiago M., Bello-Pérez L., Tecante A. (2004). Swelling-Solubility Characteristics, Granule Size Distribution and Rheological Behavior of Banana (*Musa paradisiaca*) Starch. Carbohydr. Polym..

[28] Olatunde G.O., Arogundade L.K., Orija O.I. (2017). Chemical, Functional and Pasting Properties of Banana and Plantain Starches Modified by Pre-Gelatinization, Oxidation and Acetylation. Cogent Food Agric..

[29] Cahyana Y., Wijaya E., Halimah T.S., Marta H., Suryadi E., Kurniati D. (2019). The Effect of Different Thermal Modifications on Slowly Digestible Starch and Physicochemical Properties of Green Banana Flour (*Musa acuminata colla*). Food Chem..

[30] Coulibaly S., Nemlin J., Amani N.G. (2006). Isolation and Partial Characterisation of Native Starches of New Banana and Plantain Hybrids (*Musa* spp.) in Comparison with That of Plantain Variety Orishele. Starch-Stärke.

[31] Tan X., Li X., Chen L., Xie F., Li L., Huang J. (2017). Effect of Heat-Moisture Treatment on Multi-Scale Structures and Physicochemical Properties of Breadfruit Starch. Carbohydr. Polym..

[32] Wei M., Andersson R., Xie G., Salehi S., Boström D., Xiong S. (2017). Properties of Cassava Stem Starch Being a New Starch Resource. Starch-Stärke.

[33] Zhu F. (2014). Structure, Physicochemical Properties, Modifications, and Uses of Sorghum Starch. Compr. Rev. Food Sci. Food Saf..

[34] Cahyana Y., Titipanillah R., Mardawati E., Sukarminah E., Rialita T., Andoyo R., Djali M., Hanidah I.-I., Setiasih I.S., Handarini K. (2018). Non-Starch Contents Affect the Susceptibility of Banana Starch and Flour to Ozonation. J. Food Sci. Technol..

[35] Waliszewski K.N., Aparicio M.A., Bello L.s.A., Monroy J.A. (2003). Changes of Banana Starch by Chemical and Physical Modification. Carbohydr. Polym..

[36] Bello-Pérez L.A., Romero-Manilla R., Paredes-López O. (2000). Preparation and Properties of Physically Modified Banana Starch Prepared by Alcoholic-Alkaline Treatment. Starch-Stärke.

[37] Babu D., Mohan Naik G.N., James J., Aboobacker A.B., Eldhose A., Jagan Mohan R. (2018). A Comparative Study on Dual Modification of Banana (*Musa paradisiaca*) Starch by Microwave Irradiation and Cross-Linking. J. Food Meas. Charact..

[38] Thanyapanich N., Jimtaisong A., Rawdkuen S. (2021). Functional Properties of Banana Starch (*Musa* Spp.) and Its Utilization in Cosmetics. Molecules.

[39] Bi Y., Zhang Y., Jiang H., Hong Y., Gu Z., Cheng L., Li Z., Li C. (2017). Molecular Structure and Digestibility of Banana Flour and Starch. Food Hydrocoll..

[40] Jiang H., Zhang Y., Hong Y., Bi Y., Gu Z., Cheng L., Li Z., Li C. (2015). Digestibility and Changes to Structural Characteristics of Green Banana Starch During In vitro Digestion. Food Hydrocoll..

[41] De la Torre-Gutiérrez L., Chel-Guerrero L.A., Betancur-Ancona D. (2008). Functional Properties of Square Banana (*Musa balbisiana*) Starch. Food Chem..

[42] Alimi B.A., Workneh T.S., Oke M.O. (2016). Effect of Hydrothermal Modifications on the Functional, Pasting and Morphological Properties of South African Cooking Banana and Plantain. CYTA J. Food.

[43] Khawas P., Deka S.C. (2017). Effect of Modified Resistant Starch of Culinary Banana on Physicochemical, Functional, Morphological, Diffraction, and Thermal Properties. Int. J. Food Prop..

[44] Agama-Acevedo E., Rodriguez-Ambriz S.L., García-Suárez F.J., Gutierrez-Méraz F., Pacheco-Vargas G., Bello-Pérez L.A. (2014). Starch Isolation and Partial Characterization of Commercial Cooking and Dessert Banana Cultivars Growing in Mexico. Starch-Stärke.

[45] Wang S., Li C., Copeland L., Niu Q., Wang S. (2015). Starch Retrogradation: A Comprehensive Review. Compr. Rev. Food Sci. Food Saf..

[46] Zhao K., Zhang B., Su C., Gong B., Zheng J., Jiang H., Zhang G., Li W. (2020). Repeated Heat-Moisture Treatment: A More Effectiveway for Structural and Physicochemical Modification of Mung Bean Starch Compared with Continuous Way. Food Bioproc. Technol..

[47] Vermeylen R., Goderis B., Delcour J.A. (2006). An X-Ray Study of Hydrothermally Treated Potato Starch. Carbohydr. Polym..

[48] Huang T.-T., Zhou D.-N., Jin Z.-Y., Xu X.-M., Chen H.-Q. (2016). Effect of Repeated Heat-Moisture Treatments on Digestibility, Physicochemical and Structural Properties of Sweet Potato Starch. Food Hydrocoll..

[49] Bian L., Chung H.-J. (2016). Molecular Structure and Physicochemical Properties of Starch Isolated from Hydrothermally Treated Brown Rice Flour. Food Hydrocoll..

[50] Kumar R., Khatkar B.S. (2017). Thermal, Pasting and Morphological Properties of Starch Granules of Wheat (*Triticum aestivum* L.) Varieties. J. Food Sci. Technol..

[51] Cornejo-Ramírez Y.I., Martínez-Cruz O., Del Toro-Sánchez C.L., Wong-Corral F.J., Borboa-Flores J., Cinco-Moroyoqui F.J. (2018). The Structural Characteristics of Starches and Their Functional Properties. CYTA J. Food.

[52] Lustosa B.H.B., Leonel M., Mischan M. (2009). Production of Cassava Flour Instant: The Effects of the Extrusion on the Thermal Properties and Plastic. Acta Sci. Technol..

[53] Wang J., Huang H.H., Chen P.S. (2017). Structural and Physicochemical Properties of Banana Resistant Starch from Four Cultivars. Int. J. Food Prop..

[54] Englyst H.N., Kingman S.M., Cummings J. (1992). Classification and Measurement of Nutritionally Important Starch Fractions. Eur. J. Clin. Nutr..

[55] Cummings J.H., Beatty E.R., Kingman S.M., Bingham S.A., Englyst H.N. (1996). Digestion and Physiological Properties of Resistant Starch in the Human Large Bowel. Br. J. Nutr..

[56] Štěrbová L., Bradová J., Sedláček T., Holasová M., Fiedlerová V., Dvořáček V., Smrčková P. (2016). Influence of Technological Processing of Wheat Grain on Starch Digestibility and Resistant Starch Content. Starch-Stärke.

[57] Sandhu K.S., Siroha A.K., Punia S., Nehra M. (2020). Effect of Heat Moisture Treatment on Rheological and in Vitro Digestibility Properties of Pearl Millet Starches. Carbohydr. Polym. Technol. Appl..

[58] Polesi L.F., Sarmento S.B.S., Canniatti-Brazaca S.G. (2018). Starch Digestibility and Functional Properties of Rice Starch Subjected to Gamma Radiation. Rice Sci..

[59] Toutounji M.R., Farahnaky A., Santhakumar A.B., Oli P., Butardo V.M., Blanchard C.L. (2019). Intrinsic and Extrinsic Factors Affecting Rice Starch Digestibility. Trends Food Sci. Technol..

[60] Li Z., Guo K., Lin L., He W., Zhang L., Wei C. (2018). Comparison of Physicochemical Properties of Starches from Flesh and Peel of Green Banana Fruit. Molecules.

[61] Aparicio-Saguilán A., Valera-Zaragoza M., Perucini-Avendaño M., Páramo-Calderón D.E., Aguirre-Cruz A., Ramírez-Hernández A., Bello-Pérez L.A. (2015). Lintnerization of Banana Starch Isolated from Underutilized Variety: Morphological, Thermal, Functional Properties, and Digestibility. CYTA J. Food.

[62] Gallant D.J., Bouchet B., Buléon A., Perez S. (1992). Physical Characteristics of Starch Granules and Susceptibility to Enzymatic Degradation. Eur. J. Clin. Nutr..

[63] Zavareze E.d.R., Dias A.R.G. (2011). Impact of Heat-Moisture Treatment and Annealing in Starches: A Review. Carbohydr. Polym..

[64] Gunaratne A., Hoover R. (2002). Effect of Heat–Moisture Treatment on the Structure and Physicochemical Properties of Tuber and Root Starches. Carbohydr. Polym..

[65] Miyazaki M., Morita N. (2005). Effect of Heat-Moisture Treated Maize Starch on the Properties of Dough and Bread. Food Res. Int..

[66] Lehmann U., Robin F. (2007). Slowly Digestible Starch—Its Structure and Health Implications: A Review. Trends Food Sci. Technol..

[67] BeMiller J.N., Huber K.C. (2015). Physical Modification of Food Starch Functionalities. Annu. Rev. Food Sci. Technol..

[68] De la Rosa-Millán J., Agama E., Osorio-Díaz P., Bello-Pérez L.A. (2014). Effect of Cooking, Annealing and Storage on Starch Digestibility and Physicochemical Characteristics of Unripe Banana Flour. Rev. Mex. Ing. Qumica.

[69] Loypimai P., Moongngarm A. (2015). Utilization of Pregelatinized Banana Flour as a Functional Ingredient in Instant Porridge. J. Food Sci. Technol..

[70] Azaripour A., Abbasi H. (2019). Effect of Type and Amount of Modified Corn Starches on Qualitative Properties of Low-Protein Biscuits for Phenylketonuria. Food Sci. Nutr..

[71] Chen J.-Y., Liu J., Tang X., Shen X., Liu S. (2017). Correlations between the Physical Properties and Chemical Bonds of Extruded Corn Starch Enriched with Whey Protein Concentrate. RSC Adv..

[72] Liu Y., Chen J., Luo S., Li C., Ye J., Liu C., Gilbert R.G. (2017). Physicochemical and Structural Properties of Pregelatinized Starch Prepared by Improved Extrusion Cooking Technology. Carbohydr. Polym..

[73] Awolu O., Odoro J., Adeloye J., Lawal O. (2020). Physicochemical Evaluation and Fourier Transform Infrared Spectroscopy Characterization of Quality Protein Maize Starch Subjected to Different Modifications. J. Food Sci..

[74] Nawaz H., Wahed R., Nawaz M., Shahwar D., Emeje M. (2020). Physical and Chemical Modifications in Starch Structure and Reactivity. Chemical Properties of Starch.

[75] BeMiller J.N., BeMiller J.N. (2019). Starches: Conversions, Modifications, and Uses. Carbohydrate Chemistry for Food Scientists.

[76] Chagam K.R., Haripriya S., Sundaramoorthy H. (2014). Effect of Acetylation on Morphology, Pasting and Functional Properties of Starch from Banana (*Musa AAB*). Indian J. Sci. Res..

[77] Dumancela K., Mayorga Llerena E., Santamaría J. (2020). Acetylation of Starch Extracted from Rejected Fruits of *Musa* × *Paradisiaca* L. to Obtain a Pharmaceutical Disintegrant. Pharm. Pharmacol..

[78] Singh H., Sodhi N.S., Singh N. (2012). Structure and Functional Properties of Acetylated Sorghum Starch. Int. J. Food Prop..

[79] Salcedo J., RuyDiaz J., Quintero A. (2016). Effect of the Acetylation Process on Native Starches of Yam (*Dioscorea* spp.). Rev. Fac. Nac. Agron. Medellin.

[80] Trela V., Ramallo A., Albani O. (2020). Synthesis and Characterization of Acetylated Cassava Starch with Different Degrees of Substitution. Braz. Arch. Biol. Technol..

[81] Carlos-Amaya F., Osorio-Diaz P., Agama-Acevedo E., Yee-Madeira H., Bello-Perez L.A. (2011). Physicochemical and Digestibility Properties of Double-Modified Banana (*Musa paradisiaca* L.) Starches. J. Agric. Food Chem..

[82] Chávez-Murillo C.E., Wang Y.-J., Bello-Pérez L.A. (2008). Morphological, Physicochemical and Structural Characteristics of Oxidized Barley and Corn Starches. Starch-Stärke.

[83] Bajaj R., Singh N., Kaur A. (2019). Properties of Octenyl Succinic Anhydride (Osa) Modified Starches and Their Application in Low Fat Mayonnaise. Int. J. Biol. Macromol..

[84] Bello-Pérez L., Bello-Flores C., Núñez-Santiago M., Coronel-Aguilera C., Alvarez-Ramirez J. (2015). Effect of the Degree of Substitution of Octenyl Succinic Anhydride-Banana Starch on Emulsion Stability. Carbohydr. Polym..

[85] Yu Z.-Y., Jiang S.-W., Zheng Z., Cao X.-M., Hou Z.-G., Xu J.-J., Wang H.-L., Jiang S.-T., Pan L.-J. (2019). Preparation and Properties of Osa-Modified Taro Starches and Their Application for Stabilizing Pickering Emulsions. Int. J. Biol. Macromol..

[86] Chen Y.-F., Kaur L., Singh J., Sjöö M., Nilsson L. (2018). Chemical Modification of Starch. Starch in Food.

[87] Lee J.-S., Chin-Shin Loh P., George R., Yusoff N.F. (2021). Optimization of Reaction Conditions for Hydroxypropylation of Saba Banana Starch. J. Adv. Res. Fluid Mech. Therm. Sci..

[88] Lawal O.S. (2009). Starch Hydroxyalkylation: Physicochemical Properties and Enzymatic Digestibility of Native and Hydroxypropylated Finger Millet (*Eleusine Coracana*) Starch. Food Hydrocoll..

[89] Sheng L., Wu S., Zhang H., Jiang X. (2012). Synthesis of Hydroxypropyl Dioscorea Alata Starch Rapidly Prepared with Ultrasonic-Microwave Assistance. Adv. Mat. Res..

[90] Liu H., Li M., Chen P., Yu L., Chen L., Tong Z. (2010). Morphologies and Thermal Properties of Hydroxypropylated High-Amylose Corn Starch. Cereal Chem..

[91] Olayinka F., Olayinka O., Olu-Owolabi B., Adebowale K.O. (2015). Effect of Chemical Modifications on Thermal, Rheological and Morphological Properties of Yellow Sorghum Starch. J. Food Sci. Technol..

[92] Lawal O.S. (2005). Studies on the Hydrothermal Modifications of New Cocoyam (*Xanthosoma sagittifolium*) Starch. Int. J. Biol. Macromol..

[93] Karim A.A., Nadiha M.Z., Chen F.K., Phuah Y.P., Chui Y.M., Fazilah A. (2008). Pasting and Retrogradation Properties of Alkali-Treated Sago (*Metroxylon sagu*) Starch. Food Hydrocoll..

[94] Kim H.-S., Choi H.-S., Kim B.-Y., Baik M.-Y. (2011). Ultra High Pressure (Uhp)-Assisted Hydroxypropylation of Corn Starch. Carbohydr. Polym..

[95] Hazarika B.J., Sit N. (2016). Effect of Dual Modification with Hydroxypropylation and Cross-Linking on Physicochemical Properties of Taro Starch. Carbohydr. Polym..

[96] Oladebeye A., Oshodi A., Amoo I.A., Karim A. (2013). Hydroxypropyl Derivatives of Legume Starches: Functional, Rheological and Thermal Properties. Starch-Stärke.

[97] Fu Z., Chen J., Luo S.-J., Liu C.-M., Liu W. (2015). Effect of Food Additives on Starch Retrogradation: A Review. Starch-Stärke.

[98] Orsuwan A., Sothornvit R. (2015). Effect of Miniemulsion Cross-Linking and Ultrasonication on Properties of Banana Starch. Int. J. Food Sci. Technol..

[99] Sánchez-Rivera M.M., García-Suárez F.J.L., Velázquez del Valle M., Gutierrez-Meraz F., Bello-Pérez L.A. (2005). Partial Characterization of Banana Starches Oxidized by Different Levels of Sodium Hypochlorite. Carbohydr. Polym..

[100] Cahyana Y., Pratiwi P.A., Marta H., Djali M., Halim I.R., Urrohmah S., Khairunnissa D.S., Sutardi A.A. (2019). Oxidation by Hydrogen Peroxide Changes Crystallinity and Physicochemical Properties of Banana Flour. IOP Conf. Ser. Earth Environ. Sci..

[101] Naknaen P., Tobkaew W., Chaichaleom S. (2017). Properties of Jackfruit Seed Starch Oxidized with Different Levels of Sodium Hypochlorite. Int. J. Food Prop..

[102] Obadi M., Zhu K.-X., Peng W., Sulieman A.A., Mohammed K., Zhou H.-M. (2018). Effects of Ozone Treatment on the Physicochemical and Functional Properties of Whole Grain Flour. J. Cereal Sci..

[103] Halal S.L.M.E., Colussi R., Pinto V.Z., Bartz J., Radunz M., Carreño N.L.V., Dias A.R.G., Zavareze E.d.R. (2015). Structure, Morphology and Functionality of Acetylated and Oxidised Barley Starches. Food Chem..

[104] Li S., Ward R., Gao Q. (2011). Effect of Heat-Moisture Treatment on the Formation and Physicochemical Properties of Resistant Starch from Mung Bean (*Phaseolus radiatus*) Starch. Food Hydrocoll..

[105] Olayinka O.O., Adebowale K.O., Olu-Owolabi B.I. (2008). Effect of Heat-Moisture Treatment on Physicochemical Properties of White Sorghum Starch. Food Hydrocoll..

[106] Xie H., Gao J., Xiong X., Gao Q. (2018). Effect of Heat-Moisture Treatment on the Physicochemical Properties and In Vitro Digestibility of the Starch-Guar Complex of Maize Starch with Varying Amylose Content. Food Hydrocoll..

[107] Jayakody L., Hoover R. (2008). Effect of Annealing on the Molecular Structure and Physicochemical Properties of Starches from Different Botanical Origins—A Review. Carbohydr. Polym..

[108] Björck I., Liljeberg H., Ostman E. (2000). Low Glycemic Index Foods. Br. J. Nutr..

[109] Quintero-Castaño V.D., Vasco-Leal J.F., Cuellar-Nuñez L., Luzardo-Ocampo I., Castellanos-Galeano F., Álvarez-Barreto C., Bello-Pérez L.A., Cortés-Rodriguez M. (2021). Novel Osa-Modified Starch from Gros Michel Banana for Encapsulation of Andean Blackberry Concentrate: Production and Storage Stability. Starch-Stärke.

[110] Zheng Z., Stanley R., Gidley M., Dhital S. (2015). Structural Properties and Digestion of Green Banana Flour as a Functional Ingredient in Pasta. Food Funct..

[111] Ovando-Martinez M., Sáyago-Ayerdi S., Agama-Acevedo E., Goñi I., Bello-Pérez L.A. (2009). Unripe Banana Flour as an Ingredient to Increase the Undigestible Carbohydrates of Pasta. Food Chem..

[112] Noor Aziah A.A., Ho L.H., Noor Shazliana A.A., Bhat R. (2012). Quality Evaluation of Steamed Wheat Bread Substituted with Green Banana Flour. Int. Food Res. J..

[113] Aparicio-Saguilán A., Sayago-Ayerdi S., Vargas-Torres A., Tovar J., Ascencio-Otero T., Bello-Pérez L. (2007). Slowly Digestible Cookies Prepared from Resistant Starch-Rich Lintnerized Banana Starch. J. Food Compos. Anal..

[114] Cahyana Y., Rangkuti A., Siti Halimah T., Marta H., Yuliana T. (2020). Application of Heat-Moisture-Treated Banana Flour as Composite Material in Hard Biscuit. CYTA J. Food.

[115] Pelissari F., Andrade-Mahecha M., Sobral P., Menegalli F.C. (2017). Nanocomposites Based on Banana Starch Reinforced with Cellulose Nanofibers Isolated from Banana Peels. J. Colloid Interface Sci..

[116] Viana E.B.M., Oliveira N.L., Ribeiro J.S., Almeida M.F., Souza C.C.E., Resende J.V., Santos L.S., Veloso C.M. (2022). Development of Starch-Based Bioplastics of Green Plantain Banana (*Musa paradisiaca* L.) Modified with Heat-Moisture Treatment (HMT). Food Packag. Shelf Life.

[117] Wang J., Euring M., Ostendorf K., Zhang K. (2022). Biobased Materials for Food Packaging. J. Bioresour. Bioprod..

[118] Restrepo A.E., Rojas J.D., García O.R., Sánchez L.T., Pinzón M.I., Villa C.C. (2018). Mechanical, Barrier, and Color Properties of Banana Starch Edible Films Incorporated with Nanoemulsions of Lemongrass (*Cymbopogon Citratus*) and Rosemary (*Rosmarinus officinalis*) Essential Oils. Food Sci. Technol. Int..

[119] Taweechat C., Wongsooka T., Rawdkuen S. (2021). Properties of Banana (*Cavendish* spp.) Starch Film Incorporated with Banana Peel Extract and Its Application. Molecules.

[120] Sartori T., Menegalli F.C. (2016). Development and Characterization of Unripe Banana Starch Films Incorporated with Solid Lipid Microparticles Containing Ascorbic Acid. Food Hydrocoll..

[121] Medeiros Silva V.D., Coutinho Macedo M.C., Rodrigues C.G., Neris dos Santos A., de Freitas e Loyola A.C., Fante C.A. (2020). Biodegradable Edible Films of Ripe Banana Peel and Starch Enriched with Extract of Eriobotrya Japonica Leaves. Food Biosci..

[122] Pongsuwan C., Boonsuk P., Sermwittayawong D., Aiemcharoen P., Mayakun J., Kaewtatip K. (2022). Banana Inflorescence Waste Fiber: An Effective Filler for Starch-Based Bioplastics. Ind. Crops Prod..

[123] García-Ramón J.A., Carmona-García R., Valera-Zaragoza M., Aparicio-Saguilán A., Bello-Pérez L.A., Aguirre-Cruz A., Alvarez-Ramirez J. (2021). Morphological, Barrier, and Mechanical Properties of Banana Starch Films Reinforced with Cellulose Nanoparticles from Plantain Rachis. Int. J. Biol. Macromol..

[124] Silvia R.C., Angel M.F., Alejandro A.S., Rodrigo N.C., Aurelio R.H., José Eduardo B.G., Páramo Calderón D.E. (2021). Modification of Banana Starch (*Musa paradisiaca* L.) with Polyethylene Terephthalate: Virgin and Bottle Waste. Carbohydr. Res..

[125] Ramírez-Hernández A., Aparicio-Saguilán A., Reynoso-Meza G., Carrillo-Ahumada J. (2017). Multi-Objective Optimization of Process Conditions in the Manufacturing of Banana (*Musa paradisiaca* L.) Starch/Natural Rubber Films. Carbohydr. Polym..

[126] Wang X., Huang L., Zhang C., Deng Y., Xie P., Liu L., Cheng J. (2020). Research Advances in Chemical Modifications of Starch for Hydrophobicity and Its Applications: A Review. Carbohydr. Polym..

[127] Azmin S.N.H.M., Hayat N.A.b.M., Nor M.S.M. (2020). Development and Characterization of Food Packaging Bioplastic Film from Cocoa Pod Husk Cellulose Incorporated with Sugarcane Bagasse Fibre. J. Bioresour. Bioprod..

[128] Oyeoka H.C., Ewulonu C.M., Nwuzor I.C., Obele C.M., Nwabanne J.T. (2021). Packaging and Degradability Properties of Polyvinyl Alcohol/Gelatin Nanocomposite Films Filled Water Hyacinth Cellulose Nanocrystals. J. Bioresour. Bioprod..

[129] Zhang L., Zhao J., Zhang Y., Li F., Jiao X., Li Q. (2021). The Effects of Cellulose Nanocrystal and Cellulose Nanofiber on the Properties of Pumpkin Starch-Based Composite Films. Int. J. Biol. Macromol..

[130] Wang Y., Qu Q., Gao S., Tang G., Liu K., He S., Huang C. (2019). Biomass Derived Carbon as Binder-Free Electrode Materials for Supercapacitors. Carbon.

[131] Kasturi P.R., Ramasamy H., Meyrick D., Sung Lee Y., Kalai Selvan R. (2019). Preparation of Starch-Based Porous Carbon Electrode and Biopolymer Electrolyte for All Solid-State Electric Double Layer Capacitor. J. Colloid Interface Sci..

[132] Kumar S., Singh Dhapola P., Pandey S.P., Singh P.K., Chauhan M. (2021). Corn-Starch Based Porous Carbon and IL Based Electrolyte for High Efficient Supercapacitor. Mater. Today Proc..

